# Supplementary material for: Neuropathologically validated MRI to tau PET synthesis via Covariate-modulated attention networks
Source: bioRxiv. 2025 Dec 27:2025.12.27.696668. Preprint. [Version 1] doi: 10.64898/2025.12.27.696668 (PMC12776089; doi:10.64898/2025.12.27.696668)
Supplement: Supplement 1 [file media-1.pdf]

Supplementary

Figures and Tables

Table 1 Full list of regions which make up the R=32 regions of interest (ROI).

Parietal Lobe (left and right hemispherical)	Temporal Lobe (left and right hemispherical)	Other (left and right hemispherical)
Inferior Parietal, Superior Parietal, Supramarginal, Postcentral, Precuneus	Bankssts, Entorhinal, Fusiform, Superior Temporal, Middle Temporal, Inferior Temporal, Transverse Temporal, Temporal Pole, Parahippocampal	Hippocampus, Amygdala

Table 2 Cross-validation performance of alternative Deep Learning architectures in Mean Absolute Error (MAE), Mean Absolute Percentage Error (MAPE), Structural Similarity Index (SSIM), Average Region of Interest (ROI) Correlation ($Corr_{AVG}$.), and mean ROI MAPE. The Attention UNet model generally performs the best, achieving the highest SSIM, lowest MAE, second-lowest mean ROI MAPE, and second highest Average ROI correlation.

**Model**	**MAE (std)**	**MAPE (std)**	**SSIM (std)**	**$Corr_{AVG}$ (std)**	**Mean ROI MAPE (std)**
Attention UNET	0.0050 (0.0015)	3186.33 (548.14)	0.7506 (0.0933)	0.1024 (0.0394)	13.05 (1.43)
UNET	0.00888 (0.00249)	2873.20 (709.58)	0.5756 (0.2454)	0.1108 (0.0436)	24.82 (1.80)
UNETR	0.0136 (0.0053)	150753.1 (198027.5)	0.644 (0.098)	0.079 (0.033)	13.07 (1.40)
Attn UNETR	0.0159 (0.0004)	112828.2 (74154.1)	0.600 (0.017)	0.096 (0.052)	12.96 (2.21)

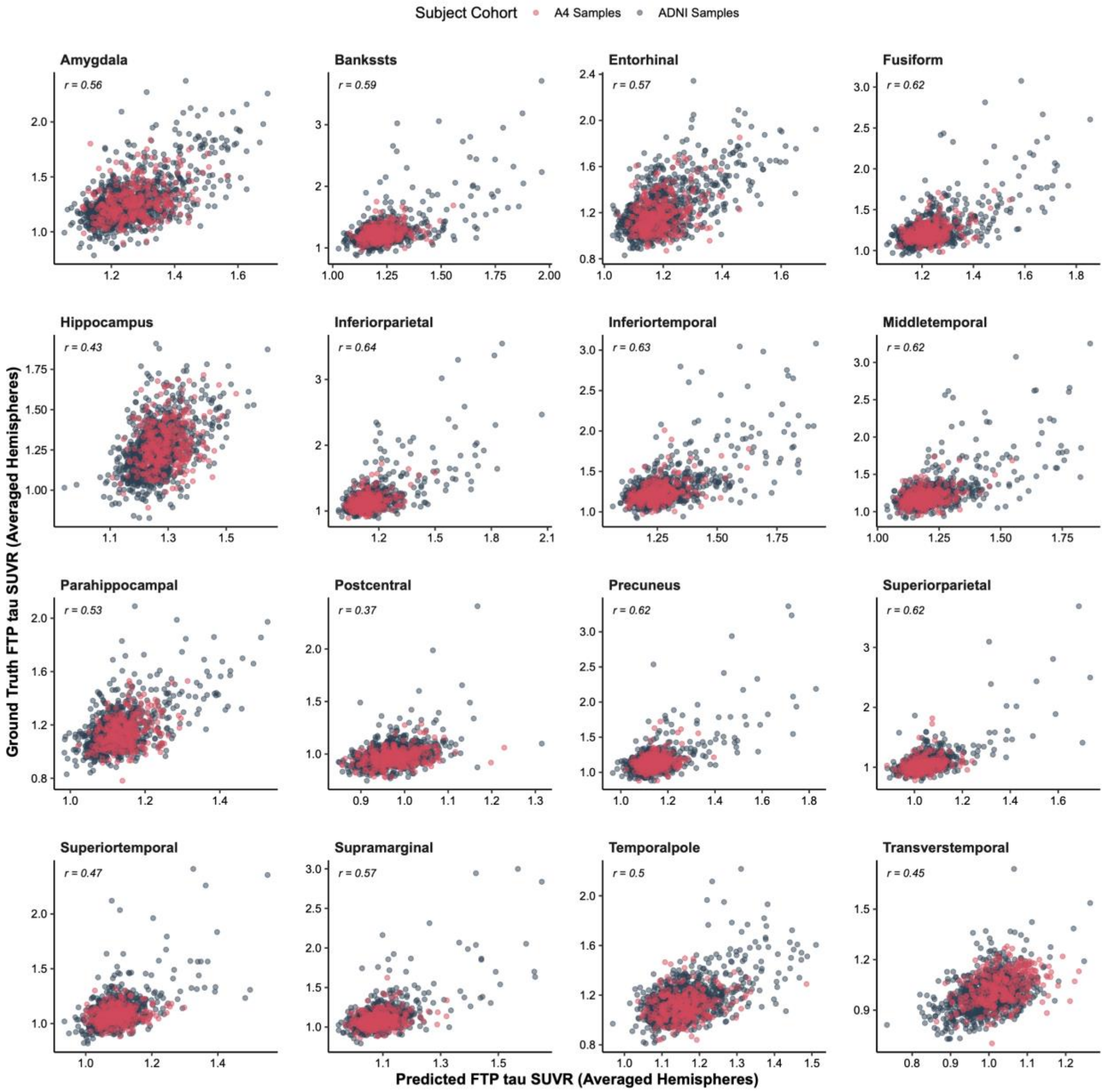

Figure 1 Hemisphere-averaged SUVR values for each ROI from ground-truth FTP tau PET versus synthetic FTP tau PET produced by Covariate-Modulated Attention UNet (CoMA-UNet), evaluated in the held-out test samples of each fold of the cross-validation splits of the ADNI-A4 training dataset. Samples from the A4-LEARN cohort are displayed with red markers, and samples from the ADNI dataset are displayed with dark-blue markers.

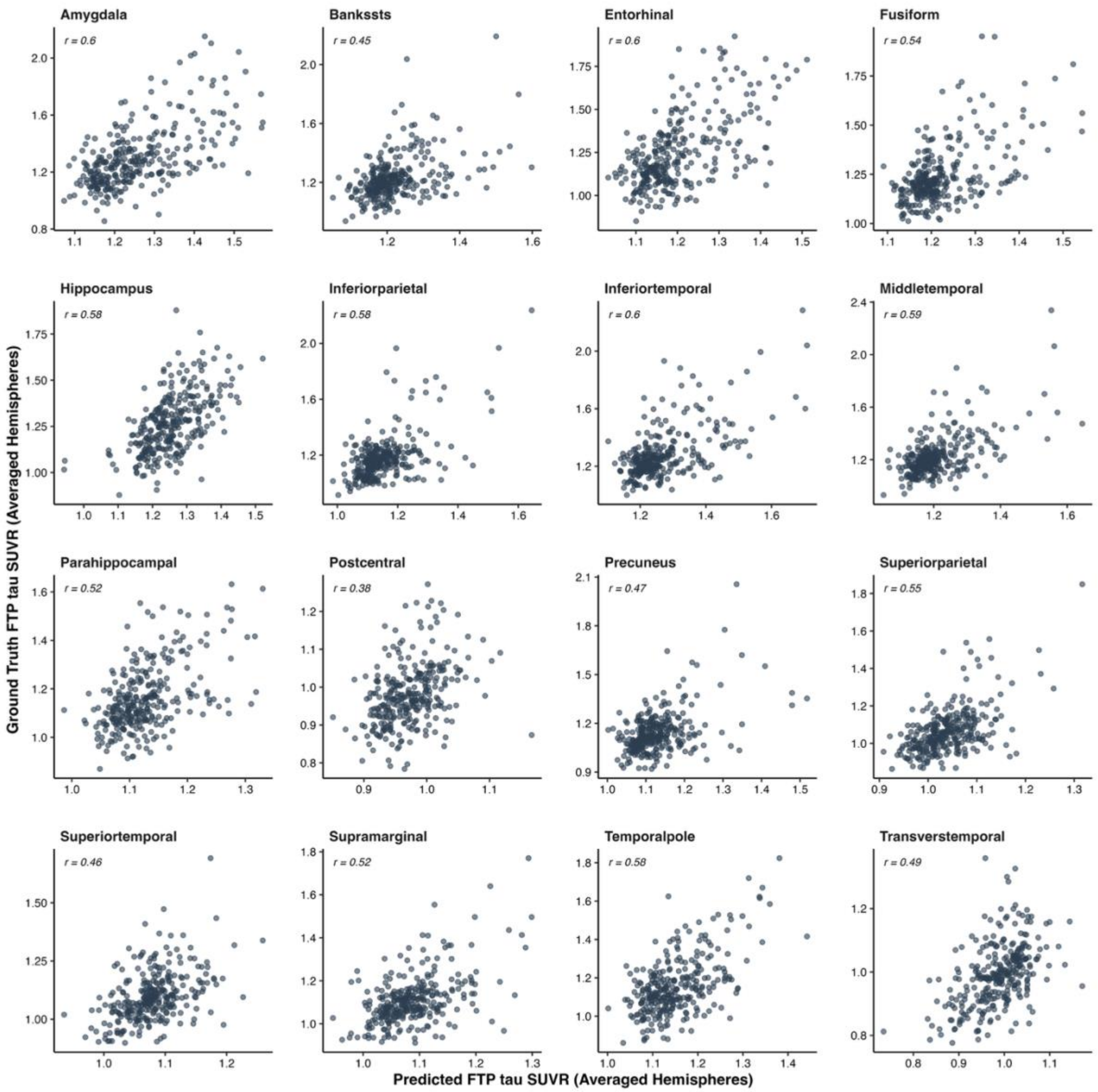

Figure 2 Point-wise comparison of synthetic hemisphere-averaged regional tau SUVR values produced by CoMA-UNet versus actual SUVR values across longitudinal FTP tau PET scans from the Longitudinal validation dataset.

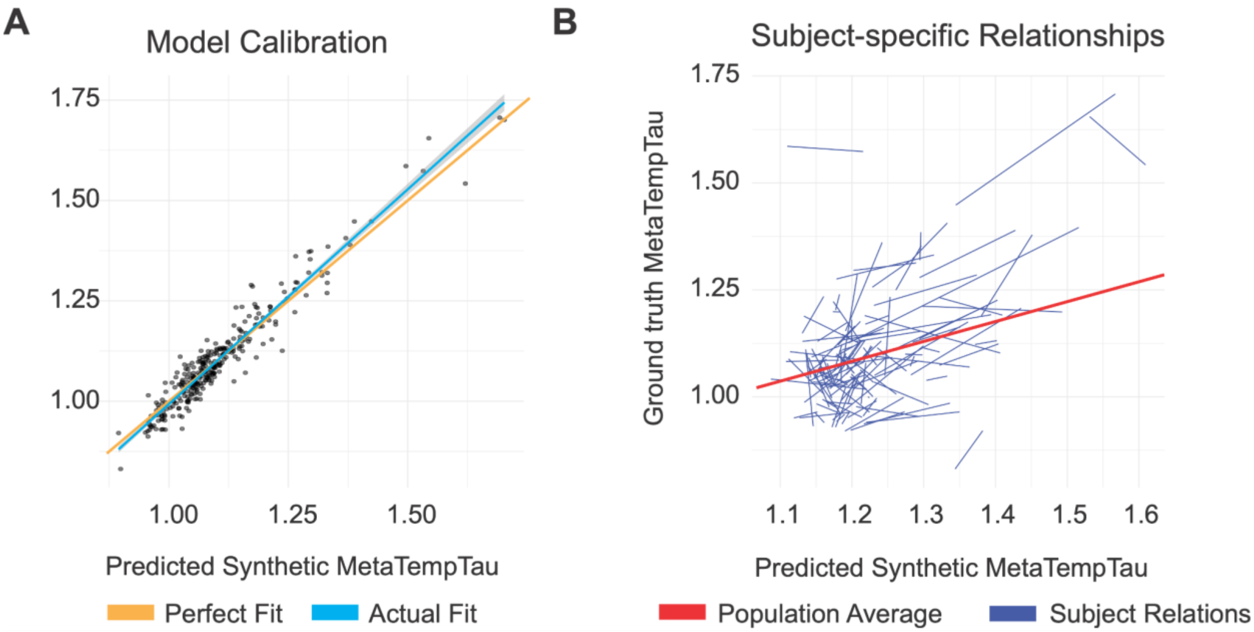

Figure 3 (A) Model calibration plot comparing linear mixed-effects model prediction using $\widehat{MetaTempTau}$ and $MetaTempTau$ across all visits within the Longitudinal validation dataset. The light blue line represents the best linear fit, and the gold line indicates the identity line. (B) Subject-specific relationships between predicted $\widehat{MetaTempTau}$ and $MetaTempTau$ (blue lines) and the population-level trajectory (red line).

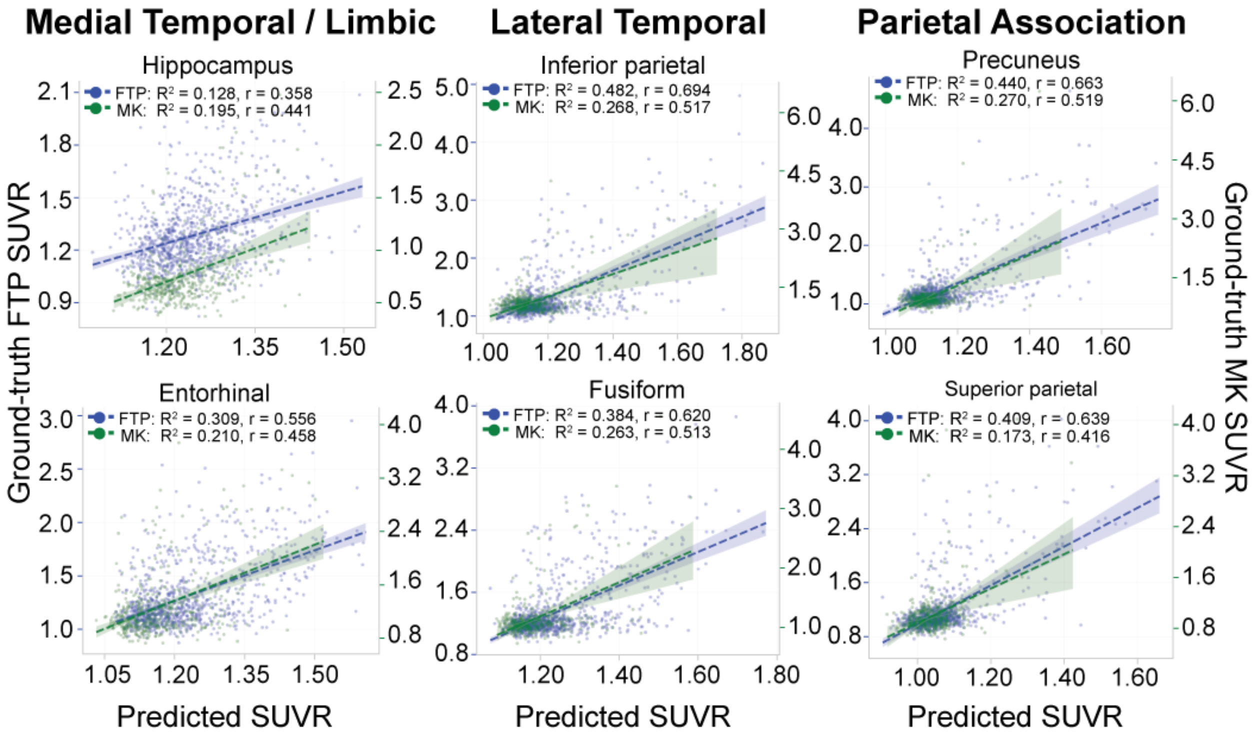

Figure 4 Correlations of synthetic tau SUVR with actual tau SUVR in key ROIs within the NACC SCAN dataset. Points corresponding to FTP tau SUVRs are displayed in blue, and points corresponding to MK-6240 tau SUVRs are displayed in green. The left-hand side y-axis marks the range of ground-truth FTP SUVRs, and the right-hand side y-axis marks the range of ground-truth MK-6240 SUVRs. Strong correlations are observed for both tracers.

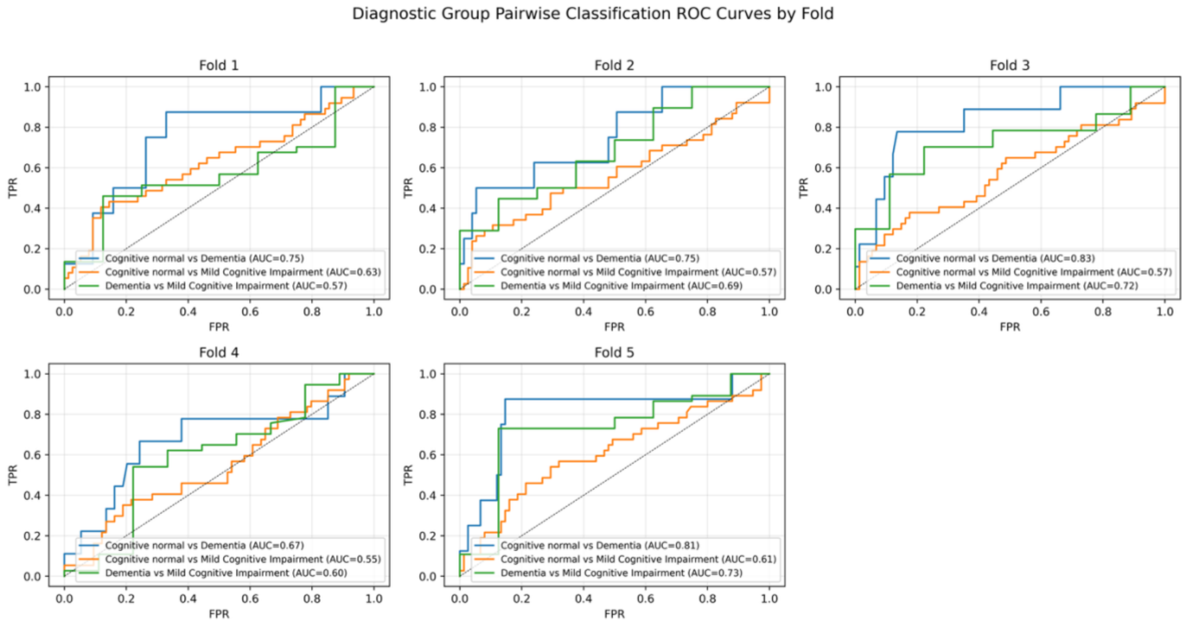

Figure 5 Diagnostic classification performance within ADNI subjects from the five-fold cross-validation of the ADNI-A4 Training Dataset, using MetaTempTau extracted from ground-truth tau PET.

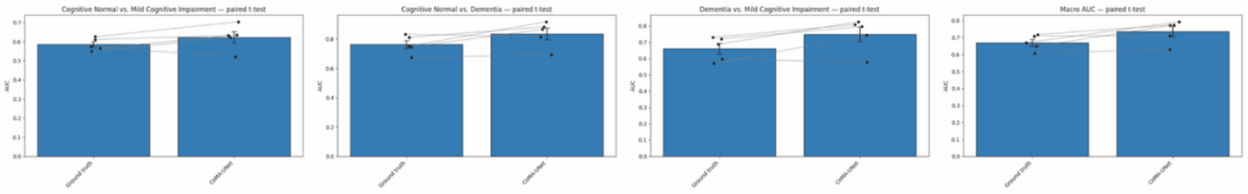

Figure 6 Comparison of diagnosis classification performance between logistic regression models using MetaTempTau extracted from actual tau PETs and synthetic tau PETs within the out-of-fold ADNI samples from the ADNI-A4 training dataset. The model using MetaTempTau from synthetic tau PETs performs better on average, and statistically the same over the five folds (paired t-test).

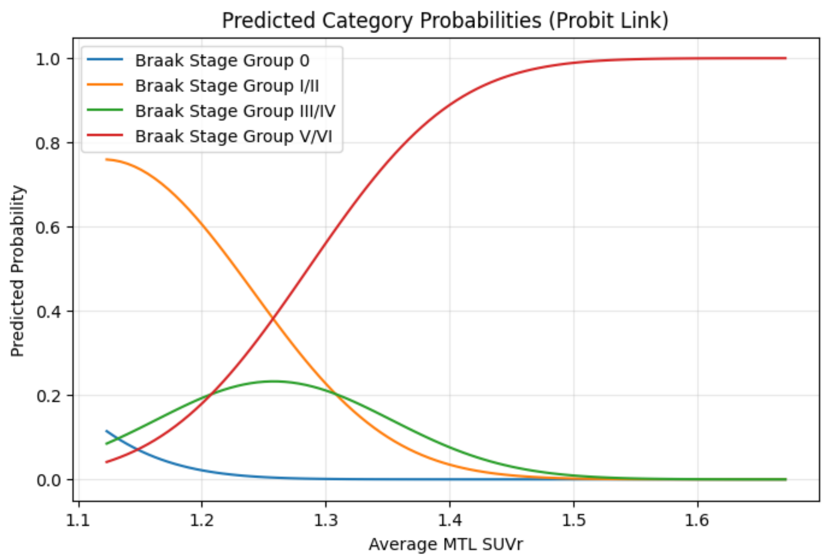

Figure 7 Ordinal regression on autopsy-confirmed Braak stage groups using average MTL SUVR from synthetic tau PETs within the ADNI-NP cohort.

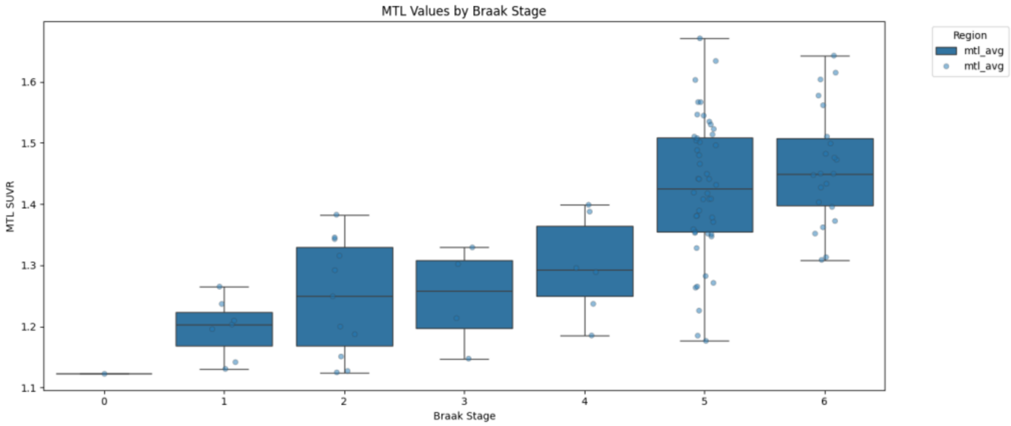

Figure 8 Average MTL SUVR by autopsy-confirmed Braak stage within the held out ADNI with available neuropath.

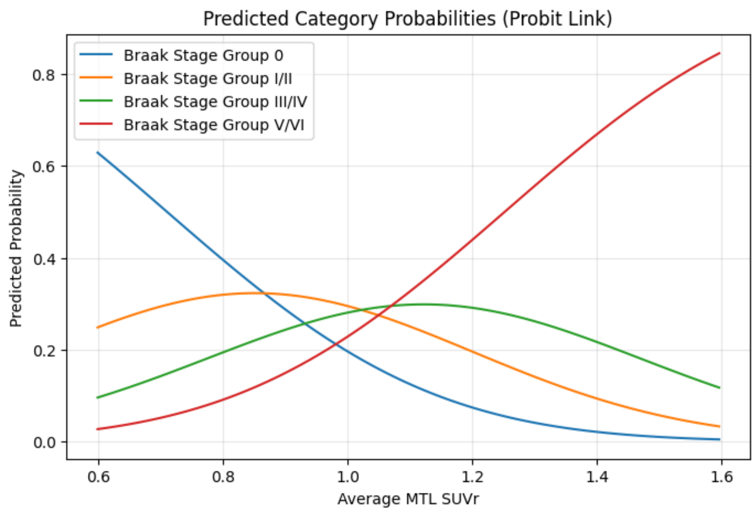

Figure 9 Ordinal regression on Braak stage group using average MTL SUVR from synthetic tau PETs within the NACC non-SCAN cohort.

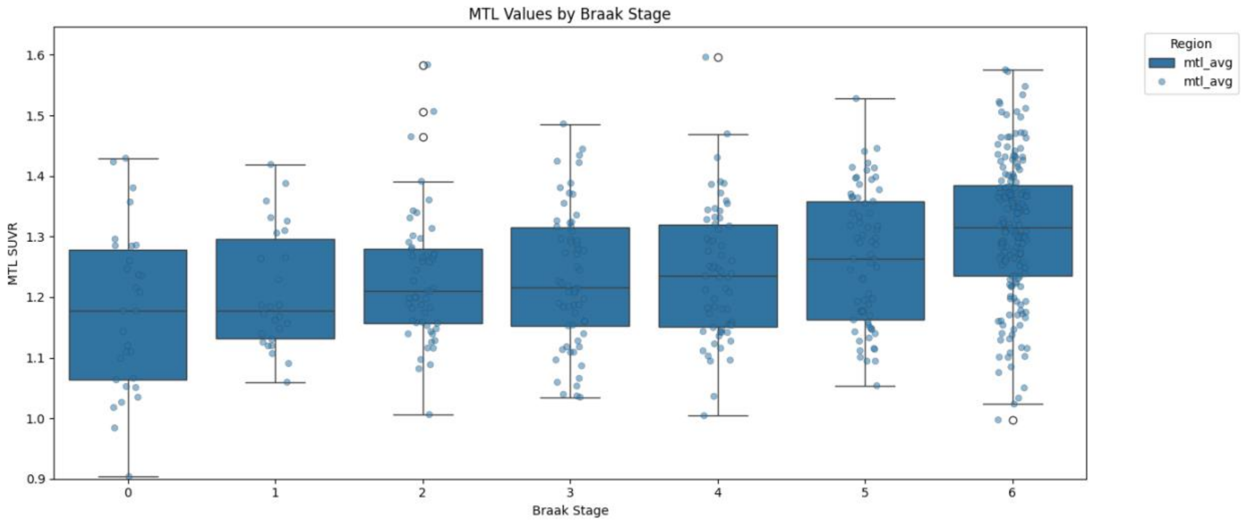

Figure 10 Average MTL SUVR by autopsy-confirmed Braak Stage within the NACC non-SCAN cohort.

Metrics

Within a cohort of patients indexed from $i = 1, \ldots, m$, let $\hat{P}_i$ be a synthetic tau PET generated from MRI M_i, and P_i be the corresponding ground-truth tau PET. We define the following validation metrics:

- Mean Absolute Error (MAE). MAE of sample j can be described as $MAE_i(\hat{P}_i, P_i) = \frac{||vec(\hat{P}_i - P_i)||_1}{p_1 \times p_2 \times p_3}$, where $vec(\cdot)$ is the vectorization operation to a tensor and $||\cdot||_1$ is the L_1 norm. The overall MAE across the subject cohort is $MAE = \frac{1}{n}\sum_{j=1}^{m} MAE_i(\hat{P}_{i,} P)$.
- Mean Absolute Percent Error (MAPE). MAPE of sample j can be described as: $MAPE = ||vec(\hat{P}_i - P_i)/vec(P_i)||_1 \times 100\%$, where "/" is the element-wise division. The overall MAPE is thus defined as $MAPE = \frac{1}{m}\sum_{j=1}^{m} MAPE_i(\hat{P}_i, P_i)$.
- Region of Interest Correlation ($Corr_k$). We define an index matrix for a brain region of interest $R_1, \ldots, R_r \in \mathbb{R}^{p_1 \times p_2 \times p_3}$ with $r = 32$. For the k-th ROI and an arbitrary PET scan P we define the mean SUVR as $SUVR_k(P) = \frac{||vec(P \odot R_k)||_1}{||R_k||_0}$. Then, for the k-th ROI across samples $j = 1, \ldots, m$, we generate two vectors: $V_{k,Pred} = \left(SUVR_k(\hat{P}_1), \ldots, SUVR_k(\hat{P}_m)\right)$ and $V_{k,True} = \left(SUVR_k(P_1), \ldots, SUVR_k(P_m)\right)$. Finally, the correlation for the k-th ROI is calculated as: $Corr_k = Corr(V_{k,Pred}, V_{k,True})$.
- Averaged ROI Correlation ($Corr_{AVG}$). Using the definition for ROI Correlation, we define $Corr_{AVG} = \frac{1}{r}\sum_{k=1}^{r} Corr_k$.
